# Selective delivery of PLXDC1 small interfering RNA to endothelial cells for anti-angiogenesis tumor therapy using CD44-targeted chitosan nanoparticles for epithelial ovarian cancer

**DOI:** 10.1080/10717544.2018.1480672

**Published:** 2018-06-11

**Authors:** Ga Hee Kim, Ji Eun Won, Yeongseon Byeon, Min Gi Kim, Tae In Wi, Jae Myeong Lee, Yun-Yong Park, Jeong-Won Lee, Tae Heung Kang, In Duk Jung, Byung Cheol Shin, Hyung Jun Ahn, Young Joo Lee, Anil K. Sood, Hee Dong Han, Yeong-Min Park

**Affiliations:** a Department of Immunology School of Medicine, Konkuk University, Chungju, South Korea;; b Asan Institute for Life Sciences, Asan Medical Center, University of Ulsan College of Medicine, Seoul, Republic of Korea;; c Department of Convergence Medicine, University of Ulsan College of Medicine, Seoul, Republic of Korea;; d Department of Obstetrics and Gynecology, Samsung Medical Center, Sungkyunkwan University School of Medicine, Seoul, Republic of Korea;; e Bio & Drug Discovery Division, Korea Research Institute of Chemical Technology, Daejeon, Republic of Korea;; f Center for Theragnosis, Biomedical Research Institute, Korea Institute of Science and Technology, Seoul, Republic of Korea;; g Department of Bioscience and Biotechnology, Sejong University, Kwang-Jin-Gu, Seoul, Republic of Korea;; h Department of Gynecologic Oncology and Reproductive Medicine, The University of Texas MD Anderson Cancer Center, Houston, TX, USA;; i Department of Cancer Biology, The University of Texas MD Anderson Cancer Center, Houston, TX, USA;; j Center for RNA Interference and Non-coding RNA, The University of Texas MD Anderson Cancer Center, Houston, TX, USA

**Keywords:** Chitosan nanoparticles, PLXDC1 siRNA, angiogenesis tumor therapy, epithelial ovarian cancer, targeted delivery

## Abstract

Angiogenesis plays an essential role in the growth and metastasis of tumor cells, and the modulation of angiogenesis can be an effective approach for cancer therapy. We focused on silencing the angiogenic gene PLXDC1 as an important factor for anti-angiogenesis tumor therapy. Herein, we developed PLXDC1 small interfering siRNA (siRNA)-incorporated chitosan nanoparticle (CH-NP/siRNA) coated with hyaluronic acid (HA) to target the CD44 receptor on tumor endothelial cells. This study aimed to improve targeted delivery and enhance therapeutic efficacy for tumor anti-angiogenesis. The HA-CH-NP/siRNA was 200 ± 10 nm in size with a zeta potential of 26.4 mV. The loading efficiency of siRNA to the HA-CH-NP/siRNA was up to 60%. The selective binding of HA-CH-NP/siRNA to CD44-positive tumor endothelial cells increased by 2.1-fold compared with that of the CD44 nontargeted CH-NP/siRNA. PLXDC1 silencing by the HA-CH-NP/siRNA significantly inhibited tumor growth in A2780 tumor-bearing mice compared with that in the control group (*p* < .01), and mRNA expression of PLXDC1 was significantly reduced in the HA-CH-NP/siRNA-treated group. Furthermore, treatment with HA-CH-NP/siRNA resulted in significant inhibition of cell proliferation (*p* < .001), reduced microvessel density (*p* < .001), and increased cell apoptosis (*p* < .001). This study demonstrates that HA-CH-NP/siRNA is a highly selective delivery platform for siRNA, and has broad potential to be used in anti-angiogenesis tumor therapy.

## Introduction

Angiogenesis is known to be essential for tumor growth and metastasis (Bergers & Benjamin, 2003; Weis & Cheresh, 2011). Anti-angiogenesis strategies have been investigated intensively as effective cancer therapies using monoclonal antibody-based systems such as bevacizumab (Avastin^®^) or tyrosine kinase inhibitor-based sorafenib (Nexavar^®^) and sunitinib (Sutent^®^) (Van Cutsem et al., 2012; Ramjiawan et al., 2017). Although such therapies are modestly effective, they have side effects including bleeding, arterial clots, hypertension, bowel perforation, and wound disruption (Verheul & Pinedo, 2007; Chen & Cleck, 2009; Cook & Figg, 2010). Therefore, novel methods with differential strategies are needed to improve the efficacy and safety of anti-angiogenesis tumor therapy. We selected PLXDC1, which is overexpressed in tumor endothelial cells, for the present study. PLXDC1 is closely involved in the acceleration of cell migration and invasion (Fuchs et al., 2007; Zhang et al., 2015). For targeting PLXDC1 in endothelial cells, we considered siRNA-based approaches, because it is difficult to target it with other conventional approaches (Devi, 2006; Daka & Peer, 2012; Lam et al., 2015). For delivery of siRNA, we developed a nanoparticle (NP) system because free siRNA is rapidly degraded by nucleases after administration (Layzer et al., 2004; Urban-Klein et al., 2005).

We selected chitosan (CH) for the present study, because it is nontoxic, biodegradable, and biocompatible (Seo et al., 2009; Han et al., 2010). Furthermore, CH is positively charged, and can form complexes with anionic siRNAs through electrostatic interactions (Lallana et al., 2017; Naito et al., 2017). The formation of the complex enables endocytosis and intracellular penetration (Chen et al., 2014; Cho et al., 2016). The ‘spongy effect’ enables CH-NPs to efficiently release siRNA from endosomes/lysosomes to the cytoplasm (Ragelle et al., 2013). In addition, we labeled hyaluronic acid (HA) on the surface of the CH-NPs to enable targeting of the CD44 receptor on tumor endothelial cells, because HA as a ligand could specifically bind on CD44 receptor (Griffioen et al., 1997; Yang et al., 2017).

**Figure 1.**
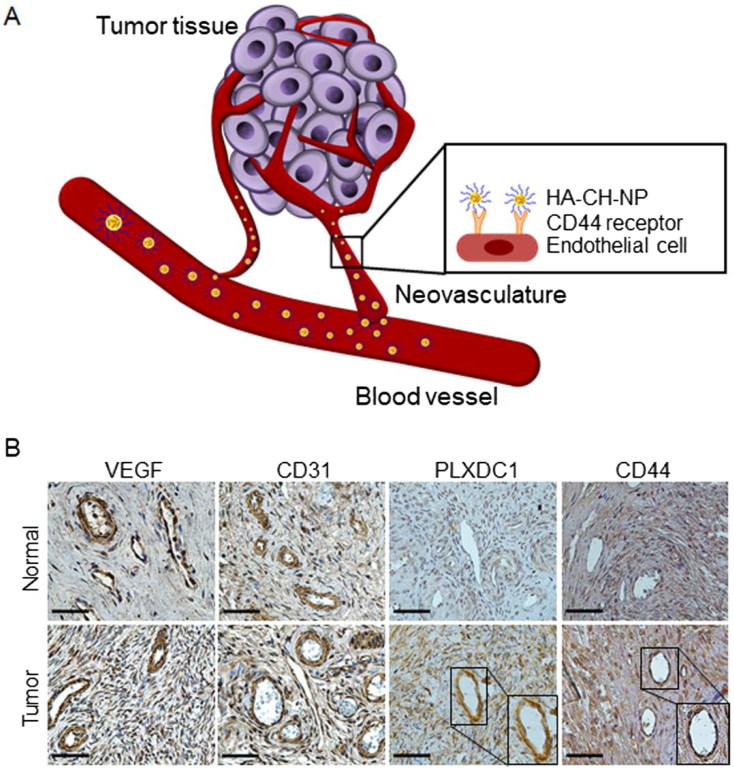
Schematic illustration of CD44-targeted delivery of HA-CH-NP/siRNA as a selective PLXDC1 siRNA delivery carrier for anti-angiogenesis tumor therapy. (A) Overview of this study. (B) Expression of PLXDC1 in normal tissues and in ovarian tissues from ovarian cancer patients. Scale bars indicate 50 µm.

In this study, we demonstrated that the HA-labeled CH-NPs incorporating PLXDC1 siRNA (HA-CH-NP/PLXDC1 siRNA) can be used for targeted siRNA delivery to the tumor vasculature (Figure 1(A)). This study demonstrated that HA-CH-NP/PLXDC1 siRNA reduces angiogenesis in tumors and, therefore, may be useful for anti-angiogenesis tumor therapy.

## Materials and methods

### Materials

Chitosan (CH, low molecular weight; deacetylation degree, 75–85%), sodium tripolyphosphate (TPP), and acetic acid were purchased from Sigma-Aldrich (St. Louis, MO). HA was purchased from Tokyo Chemical Industry (Tokyo, Japan). Control siRNA (target sequence: 5′-UUCUCCGAACGUGUCACGU-3′) and PLXDC1 siRNA (target sequence: 5′-GACACCUGCGUCCUCGA-3′) were purchased from Sigma-Aldrich (St. Louis, MO). Fetal bovine serum (FBS), penicillin–streptomycin, and Dulbecco’s modified Eagle’s medium (DMEM) were purchased from Gibco BRL/Life Technologies (New York, NY). All other materials were of analytical grade and were used without further purification.

### Cell lines and culture conditions

Human epithelial ovarian cancer cell lines A2780, HeyA8, and SKOV3 were maintained and propagated in RPMI 1640 supplemented with 10% of FBS and 0.1% of gentamicin sulfate (Xu et al., 2017; Yan et al., 2017). Human endothelial cells (HUVEC) were maintained in EBM-2 medium (Lonza, Walkersville, MD), and murine ovarian endothelial cells (MOEC) were maintained in DMEM supplemented with 4500 mg/L d-glucose, 110 mg/L sodium pyruvate, l-glutamine, and sodium bicarbonate. The cell cultures were maintained in an incubator at 37 °C and 5% CO_2_.

### Preparation of the CH-NP

The preparation of the siRNA-incorporated CH-NP (CH-NP/siRNA) depended on the electronic interaction between cationic CH, and anionic TPP and siRNA (Lu et al., 2010). Briefly, predetermined concentrations of TPP (0.25% w/v) and siRNA (1 μg/μL) were added to CH (2 mg/ml, 1% acetic acid) solution, and the CH-NP/siRNA formed spontaneously as the mixture was stirred continuously at room temperature. After incubation at 4 °C for 30 min, the CH-NP/siRNA was collected by centrifugation (Hanil Science Industrial, Korea) at 13,000 rpm for 50 min at 4 °C. After preparing the CH-NP/siRNA, we added HA (1 mg/mL) in resuspended CH-NP/siRNA solution to label the HA on the surface of the CH-NP/siRNA by electronic interaction. The HA-labeled CH-NPs/siRNA (HA-CH-NP/siRNA) was obtained by centrifugation at 13,000 rpm for 50 min at 4 °C, and was stored at 4 °C until further use. The size and zeta potential of the HA-CH-NP/siRNA were measured by light scattering with a particle size analyzer and Zeta Plus (Brookhaven Instrument Co., CA), respectively. Encapsulation efficiency and release of siRNA was measured by ND-1000 spectrophotometer (NanoDrop Technology, USA) at 260 nm using the supernatant obtained after the centrifugation of CH-NP/siRNA or HA-CH-NP/siRNA (Katas & Alpar, 2006). The morphology of the HA-CH-NP/siRNA was examined by atomic force microscopy (AFM, XE-100, Park Systems, Korea).

### Cell migration and invasion assay following PLXDC1 silencing

We performed cell migration and invasion assays to confirm the biological effect on HUVEC cells following PLXDC1 silencing with siRNA. The PLXDC1 siRNA was transfected into the HUVEC cell for 4 h in a CO_2_ incubator. After incubation, the HUVEC cells (1 × 10^5^) were collected in EBM-2 serum-free medium, and placed in the upper chamber of a Transwell support coated with 0.1% gelatin (PET track-etched membrane, Corning, NY). The chamber was placed on a 24-well plate, and an EBM-2 medium containing serum was placed in the lower chamber to act as a chemo-attractant. The cells were incubated for 6 h to allow migration. Thereafter, they were stained with 0.1% crystal violet for 30 min, and examined by light microscopy (×400 magnification).

In addition, we performed an invasion assay on HUVECs following PLXDC1 siRNA treatment. Before seeding the cells, we prepared chambers coated with 100 μL of Matrigel (BD Biosciences) at 37 °C for 4 h. HUVEC cells (5 × 10^4^) in serum-free EBM-2 medium were placed in the upper chamber of a Transwell support (PET track-etched membrane, Corning, NY). EBM-2 medium containing serum was then added to the lower chamber. After incubating for 24 h, the lower surface of the membrane was fixed with 4% paraformaldehyde and stained with 0.1% crystal violet. The cells were examined by light microscopy (×400 magnification).

### Tube formation assay

To confirm tube formation with HUVECs following PLXDC1 siRNA treatment, 50 µL of Matrigel (BD Biosciences) was quickly added to each well of a 96-well plate and allowed to solidify for 10 min at 37 °C. The HUVEC cells (10,000 per well) were then incubated. The formation of capillary-like structures was observed using an Olympus IX81 inverted microscope (×400 magnification). The capillary-like structures formed in the gel were examined by analysis of the digitized images (Rikitake et al., 2002).

### CD44-targeted binding of HA-CH-NP/siRNA

Prior to determining CD44-targeted binding, we selected CD44-positive or CD44-negative cell lines by flow cytometry (FACScan, Becton Dickinson, Mountain View, CA) using CELLQuest software (Immunocytometry Systems, Becton Dickinson). After selecting the cell lines, we assessed the CD44-targeted binding of HA-CH-NPs/siRNA by confocal microscopy (LSM 710, Carl Zeiss, Oberkochen, Germany). Briefly, we added Cy5-siRNA-incorporated CH-NPs or Cy5-siRNA-incorporated HA-CH-NPs to A2780 or MOEC, and incubated the cells for 40 min at room temperature. Thereafter, the cells were fixed using 4% paraformaldehyde for 10 min. After fixation, the cells were stained with Sytox green for 10 min at room temperature and examined by confocal microscopy.

### In vivo *delivery of HA-CH-NPs*

Female BALB/c nude mice (7 weeks old, 25 g) were purchased from Orient Co. (Seoul, South Korea). All the studies on mice were approved by the Konkuk University Institutional Animal Care and Use Committee (Ref. No. KU17188). To produce tumors, A2780 (1 × 10^6^ cells per 200 µL HBSS) cells were injected intraperitoneally (i.p.) into the mice. Each mouse was given an intravenous (i.v.) injection of CH-NPs/Cy5 siRNA and HA-CH-NPs/Cy5 siRNA (150 μg/kg) through its tail vein. The fluorescence signal from Cy5 siRNA was monitored in the tumor-bearing mice using an IVIS imaging system (IVIS Spectrum, MA) at the appropriate wavelength (λ_ex_ = 640 nm, λ_em_ = 680 nm). The emitted signals were collected using the time-correlated single-photon counting system software of the IVIS imaging system. Moreover, we detected the colocalization of HA-CH-NP and the CD44 receptor in the tumor tissues to confirm the delivery of CD44-targeted HA-CH-NP.

### Anti-tumor efficacy

To produce tumors, A2780 or HeyA8 cells (1 × 10^6^ cells per 200 µL HBSS) were injected i.p. into the mice. The mice (*n* = 10 per group, 6–7 weeks old) were monitored daily for adverse therapeutic effects, and were sacrificed when the control mice became moribund. To determine tumor growth, treatment began 1 week following the i.p. injection of the A2780 cells into the mice. CH-NP/PLXDC1 siRNA or HA-CH-NP/PLXDC1 siRNA was administered twice per week at a dose of 150 µg/kg body weight via i.v. injection. Body weight, tumor nodules, and tumor weight were recorded. The tissue specimens were fixed with either 4% paraformaldehyde or optimum cutting temperature compound (Sakura Finetek, USA), or were snap-frozen.

### Quantitative reverse-transcription polymerase chain reaction (qRT-PCR)

We performed qRT-PCR using 100 ng of total RNA isolated from treated tumor tissue using an RNeasy mini kit (Qiagen, USA, Cat No. 74106) according to the manufacturer’s instructions. Complementary DNA (cDNA) was synthesized from 1–3 μg of total RNA using a Verso cDNA kit (Thermo Scientific, Lithuania). Quantitative polymerase chain reaction (qPCR) analysis was performed in triplicate using a SYBR Green ER qPCR SuperMix Universal mix (Invitrogen, Carlsbad, CA) and a Bio-Rad reagent (Bio-Rad Laboratories, Hercules, CA). Relative quantification was done using the 2^−ΔΔCT^ method, which normalized the control for percent fold changes (Han et al., 2010; Lu et al., 2010)

### Immunohistochemical staining

Immunohistochemical (IHC) analysis of PLXDC1 expression, cell proliferation (Ki67), microvessel density (MVD, CD31), and apoptosis (TUNEL) was performed on the tumor tissue from the mice that had received i.v. injections of CH-NP/PLXDC1 siRNA or HA-CH-NP/PLXDC1 siRNA (Han et al., 2010). Five random fields in each of the tissue preparations were recorded at ×400 magnification. All the staining was quantified by two blinded investigators.

### Statistical analysis

Differences in continuous variables were analyzed using the Student’s *t*-test to compare two groups. Analysis of variance (ANOVA) was carried out to compare the differences between multiple groups. A *p* value of < .05 was considered statistically significant.

## Results

### PLXDC1 expression in ovarian cancer patients

We first determined whether PLXDC1 and CD44 expression in the tumor neovasculature of cancer patients in an effective target (Figure 1(B)). Whereas, vascular endothelium growth factor (VEGF) and CD31 (a tumor neovasculature protein) were highly expressed in both normal and tumor tissues, PLXDC1 was specifically expressed in tumor-associated endothelial cells in tumor tissues compared with normal tissues (Figure 1(B)). Additionally, CD44 was highly expressed in tumor endothelial cells compared to normal tissue (Figure 1(B)). Based on these results, we selected PLXDC1 as a potential therapeutic target in anti-angiogenesis tumor therapy. Therefore, we considered PLXDC1 to be a valid target for ovarian cancer therapy.

### Characteristics of HA-CH-NPs

**Figure 2.**
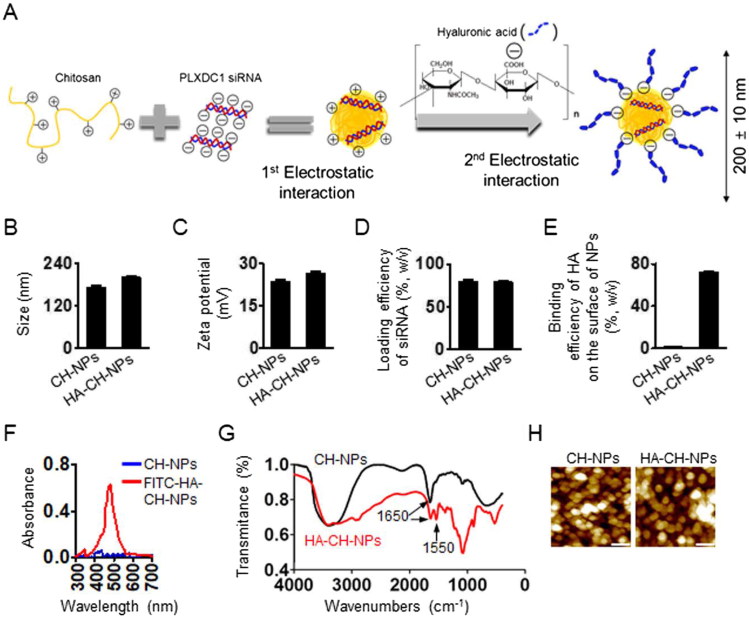
Physical properties of CH-NP/PLXDC1 siRNA and HA-CH-NP/PLXDC1 siRNA. (A) Preparation of HA-CH-NP/PLXDC1 siRNA. (B) Particle size and (C) zeta potential of HA-CH-NP/PLXDC1 siRNA. The particle size and zeta potential were measured by dynamic light scattering with a particle size analyzer. (D) The efficiency of loading Cy5-labeled control siRNA into CH-NPs and HA-CH-NPs was determined by fluorescence spectrophotometry. (E) Binding efficiency of HA on the surface of HA-CH-NPs. (F) FITC-HA-labeled with HA-CH-NPs was examined by UV-visible spectrophotometry at 494 nm. (G) The complexation of the HA-CH-NPs was determined by FT-IR spectroscopy. The FT-IR spectra of the HA-CH-NP were confirmed by amide bonds for NH vibration (N-H bending at 1650 cm^−1^) of CH and -ROOH bonding of HA at 1550 cm^−1^. (H) The morphologies of the CH-NPs and HA-CH-NPs were determined by AFM. Scale bar: 250 nm. Error bars represent SEM; *p < .05.

To target PLXDC1 as an effective therapeutic gene in tumor endothelial cells, we selected siRNA-based treatment strategy that could specifically knock down the target gene and was nontoxic to other sequences, even in normal cells (Mangala et al., 2009; Han et al., 2010). Therefore, we designed siRNA-incorporated chitosan nanoparticles (CH-NP/siRNA) to deliver siRNA effectively. In addition, we labeled CH-NP/siRNA with HA by electrostatic interaction to selectively target the CD44 receptor on tumor endothelial cells (Figure 2(A)). We first confirmed the physicochemical properties of CH-NP/siRNA and HA-CH-NP/siRNA. The size of the CH-NP/siRNA was 173 ± 10 nm [polydispersity index (PDI): 0.352], whereas the HA-CH-NP/siRNA was slightly larger (200 ± 10 nm, PDI: 0.359) owing to HA labeling (Figure 2(B)). The size distribution and PDI of the CH-NPs are shown in Supplementary Figure S1. The surface charges of CH-NPs/siRNA and HA-CH-NPs/siRNA were 23.6 ± 1.0 mV and 26.4 ± 1.0 mV, respectively (Figure 2(C)). The loading efficiency of siRNA in the CH-NP/siRNA was up to 80% and the loading efficiency of siRNA in the HA-CH-NP/siRNA was 78% (Figure 2(D)). The labeling efficiency of HA on the surface of the HA-CH-NP/siRNA was up to 72% (Figure 2(E)), and HA labeling on the HA-CH-NP/siRNA was determined by UV-vis spectroscopy at 494 nm using FITC-labeled HA (Figure 2(F)). The complexation of the HA-CH-NP was determined by Fourier Transform Infrared (FT-IR) spectroscopy (Figure 2(G)). A peak at 1650 cm^−1^ was amide I (N-H stretch) peck of CH-NPs and the peak at 1550 cm^−1^ was carboxylate (-ROOH) of HA in HA-CH-NPs (Nasti et al., 2009; Yang et al., 2012). These results indicate that CH and HA formed complexes. The morphology of the HA-CH-NP/siRNA was examined using AFM (Figure 2(H)). In addition, we assessed the release of siRNA from CH-NPs and HA-CH-NPs at pH 4 or pH 7 with maintaining the physiological body temperature (37 °C), thereby mimicking the intracellular acidic environment after cell uptake of HA-CH-NPs (Supplementary Figure S2). Although the release of siRNA from CH-NPs and HA-CH-NPs at pH7 was limited, siRNA release was increased at pH4. This result indicated that siRNA carried by CH-NP or HA-CH-NP could be readily released in an intracellular acidic environment.

### Biological effect of PLXDC1 in HUVEC cells

**Figure 3.**
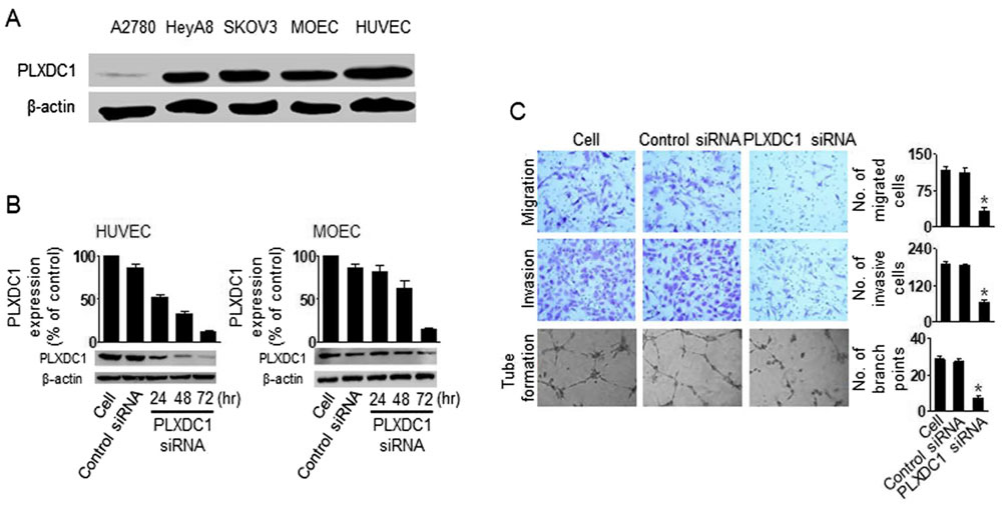
The biological effect of PLXDC1 in HUVEC cells. (A) Expression of PLXDC1 in ovarian tumor cells or endothelial cells. (B) PLXDC1 silencing using PLXDC1 siRNA in endothelial cells. (C) Invasion, migration, and tube formation in HUVEC cells following PLXDC1 silencing. Error bars represent SEM; *p < .05.

To determine the biological effects of PLXDC1 in tumor endothelial cells, we assessed migration, invasion, and tube formation after PLXDC1 silencing. Firstly, we selected PLXDC1-positive and PLXDC1-negative cells by western blotting (Figure 3(A)). The A2780 cells did not express PLXDC1, but the HUVEC and MOEC cells showed high expression of PLXDC1. Moreover, we confirmed effective gene silencing using PLXDC1 siRNA (Figure 3(B)). Finally, we assessed migration, invasion, and tube formation in the HUVEC cells following PLXDC1 silencing (Figure 3(C)). PLXDC1 silencing in the HUVEC cells resulted in significant inhibition of cell migration, invasion, and tube formation compared with the results obtained using the control siRNA (Figure 3(C)), suggesting that PLXDC1 could be an important target for anti-angiogenesis tumor therapy.

### Intracellular delivery of HA-CH-NP/siRNA

**Figure 4.**
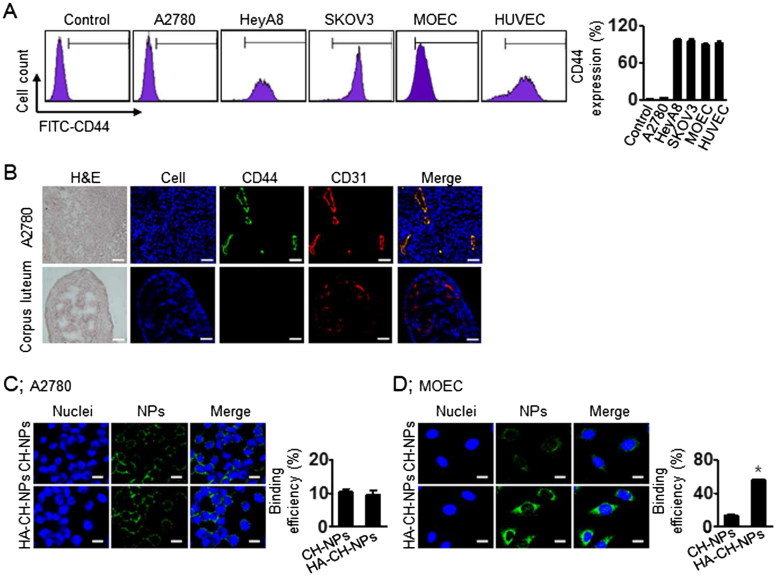
CD44-targeted delivery of HA-CH-NPs. (A) CD44 expression in ovarian tumor cells or endothelial cells. (B) CD44 expression in tumor endothelial cells against A2780 tumor tissue. CD44 (green) and tumor neovasculature protein (CD31, red) were shown in colocalization (yellow) in tumor tissue. Scale bars indicate 20 µm. (C) CD44-mediated intracellular delivery of CH-NPs and HA-CH-NPs. Scale bars indicate 10 µm. Error bars represent SEM; *p < .05.

Before performing CD44-receptor-mediated intracellular delivery of the HA-CH-NPs/siRNA, we selected CD44-positive or CD44-negative cell lines by flow cytometry (Figure 4(A)). CD44 was not expressed in the A2780 cells, whereas it was highly expressed in the HUVEC and MOEC cells (Figure 4(A)). Additionally, we confirmed CD44 expression in tumor endothelial cells from A2780 tumor tissues and compared it with the CD44 expression in the corpus luteum of mouse ovary (Figure 4(B)). Thereafter, based on CD44 expression, we determined CD44 receptor-mediated intracellular delivery of HA-CH-NP/siRNA by confocal microscopy (Figure 4(C,D) and Supplementary Figure S3). The intracellular delivery of HA-CH-NP/Cy5 siRNA was low in the A2780 cells (CD44-negative), whereas it was significantly higher in the MOEC (CD44-positive) compared with CH-NP/Cy5 siRNA delivery. These data indicate that HA-CH-NP/siRNA can specifically bind to the CD44 receptor, resulting in increased intracellular delivery.

### In vivo *delivery of HA-CH-NP/siRNA*

**Figure 5.**
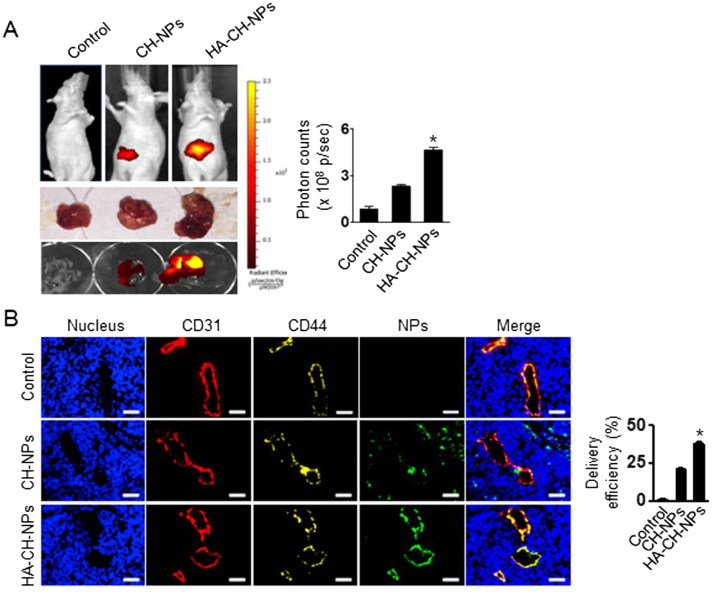
*In vivo* distribution of HA-CH-NPs/Cy5 siRNA. (A) *In vivo* distribution of HA-CH-NPs/Cy5 siRNA after i.v. injection in A2780 tumor-bearing mice. (B) HA-CH-NPs/Cy5 siRNA localization in tumor endothelial cells. The HA-CH-NPs/Cy5 siRNA was injected into mice through tail vein. The nuclei were stained with Hoechst 33258 (blue), vascular endothelial cells were stained with anti-CD31 (red), and CD44 receptors were stained with anti-CD44 (yellow). Scale bars indicate 20 µm. Error bars represent SEM; *p < .05.

We next examined the *in vivo* selective delivery of HA-CH-NP/Cy5 siRNA in A2780 tumor-bearing mice following i.v. injection, because the tumor endothelial cells were CD44-positive but the A2780 cells were CD44-negative. Tumors were induced in mice through i.p. injections of A2780 cells (1 × 10^6^ cells per 200 µL of HBSS). The HA-CH-NP/Cy5 siRNA was injected into the A2780 tumor-bearing mice, and their localization was monitored using an IVIS analyzer (Figure 5(A)). HA-CH-NP/Cy5 siRNA exhibited higher tumor localization than CH-NP/Cy5 siRNA, which might be attributed to the specific interaction between the CD44 receptor in the tumor endothelial cells and the HA-CH-NP/Cy5 siRNA. Moreover, we examined the distribution of HA-CH-NP/Cy5 siRNA in the tumor tissue following i.v. injection by confocal microscopy (Figure 5(B)). The HA-CH-NPs/Cy5 siRNA (green) was colocalized with the CD44 receptor (yellow) in the tumor neovasculature (CD31, red). This result indicated that the HA-CH-NPs/Cy5 siRNA was selectively delivered to the CD44-expressing tumor endothelial cells in the tumor tissue.

### Anti-tumor efficacy

**Figure 6.**
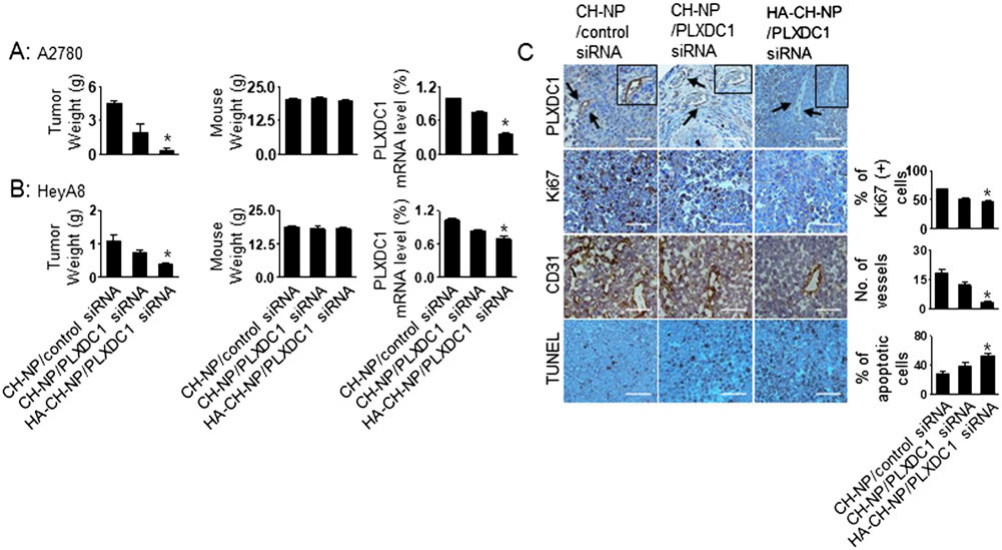
Therapeutic efficacy of HA-CH-NPs in an orthotopic ovarian cancer model. Treatment with HA-CH-NPs was started 1 week after the intraperitoneal (i.p.) injection of mice with (A) A2780 (PLXDC1-negative) and (B) HeyA8 (PLXDC1-positive) tumor cells. HA-CH-NP/PLXDC1 siRNA was injected i.v. twice per week at doses of 150 µg/kg PLXDC1 siRNA-based on body weight. The fold change in levels of PLXDC1 mRNA represents the mean of triplicates evaluated by qRT-PCR. (C) Immunohistochemical analyses for markers of PLXDC1 expression in endothelial cells (PLXDC1 antibody), cell proliferation (Ki67), microvessel density (MVD, CD31), and TUNEL were performed on A2780 tumor tissues (scale bar: 50 µm). Results represent the mean ± standard deviation (SD). Statistical tests were two-sided and *p* values were evaluated by analysis of variance (ANOVA); *p < .05.

We selected A2780 cells to demonstrate the potential therapeutic efficacy of the HA-CH-NPs. The A2780 cells did not express PLXDC1 (Figure 3(A)) or CD44 (Figure 4(A)), whereas the tumor endothelial cells showed high expression of CD44 (Figure 4(B)). Therefore, the endothelial cells in the A2780 tumor tissue expressed CD44 (Figure 5(B)), thus providing an attractive tumor model for targeting both PLXDC1 and CD44 in tumor endothelial cells. The mice were divided into the following treatment groups (*n* = 10 mice per group): (1) CH-NP/control siRNA, (2) CH-NP/mPLXDC1 siRNA, and (3) HA-CH-NP/mPLXDC1 siRNA. All the mice were euthanized when the mice in the control group became moribund. PLXDC1 siRNA (150 µg/kg) was injected i.v. twice per week. HA-CH-NP/mPLXDC1 siRNA treatment significantly inhibited tumor growth compared with CH-NP/control siRNA (91% reduced, *p* < .01) and CH-NP/mPLXDC1 siRNA (79% reduced, *p* < .03, Figure 6(A)). The weights of the mice did not differ significantly between the CH-NP and HA-CH-NP groups, which suggest that the CH-NP and HA-CH-NP were no overtly toxic with regard to therapy (Figure 6(A)). The reduction in the level of PLXDC1 mRNA was confirmed by qRT-PCR. There was a significant decrease in the level of PLXDC1 mRNA in the HA-CH-NP/mPLXDC1 siRNA treatment group compared with the CH-NP/control siRNA and CH-NP/mPLXDC1 siRNA treatment groups (Figure 6(A)). Additionally, HA-CH-NP and HA-CH-NP/control siRNA showed no therapeutic effect compared to CH-NP/control siRNA (Supplementary Figure S4). Moreover, we tested the therapeutic efficacy of the HeyA8 tumor model, which is a PLXDC1- and CD44-positive cell line (Figure 6(B)). HA-CH-NP/mPLXDC1 siRNA treatment significantly inhibited tumor growth compared with the CH-NP/control siRNA (63% reduced, *p* < .01) and CH-NP/mPLXDC1 siRNA (44% reduced, *p* < .01, Figure 6(B)) groups. There were no significant differences in total body weight (Figure 6(B)). The reduction in PLXDC1 mRNA levels was confirmed using qRT-PCR (Figure 6(B)). To determine the potential biological effects of HA-CH-NP/mPLXDC1 siRNA treatment in tumor tissues, we stained the tumors for markers of PLXDC1, cell proliferation (Ki67), microvessel density (MVD, CD31), and apoptosis (TUNEL). HA-CH-NP/mPLXDC1 siRNA treatment resulted in reduced PLXDC1 expression, inhibited cell proliferation (*p* = .001 vs. control) and MVD (*p* < .001 vs. control), and increased cell apoptosis (*p* < .001 vs. control) (Figure 6(C)). A similar effect on PLXDC1 expression, proliferation, and apoptosis was confirmed in the HeyA8 tissues compared to A2780 tumor tissues (Supplementary Figure S5).

## Discussion

We demonstrated a novel selective siRNA delivery system to tumor-associated endothelial cells using NP-based approach for anti-angiogenic therapy in epithelial ovarian carcinoma. In addition, we successfully labeled HA as a CD44 ligand on the surface of NPs by electrostatic interaction, resulting in HA-labeled CH-NP realized CD44-mediated selective delivery. This approach has broad utility for enhancing the anti-angiogenic therapeutic efficacy, which resulted in effective siRNA delivery to tumor endothelial cells and could be applied to multiple cancer models to achieve high therapeutic efficacy.

Anti-tumor angiogenesis therapy has been studied to prevent tumor metastasis and inhibit tumor growth, which is one of the effective strategies for eradicating tumor growth (Lu et al., 2012; Sennino et al., 2012). Recently, anti-angiogenic therapy using inhibitors have attracted much attention as novel therapies, and several studies have shown clinical studies (Ma et al., 2018). However, these approaches were known to be limited and had side effects (Ma et al., 2018). Therefore, a novel anti-angiogenesis therapy was needed to overcome side effects caused by inhibitor treatment.

RNAi-based cancer therapy is a highly attractive method of specific gene silencing, but hurdles related to systemic *in vivo* delivery of siRNA need to be overcome for realizing its full potential in clinical settings (Aagaard & Rossi, 2007). Moreover, delivery efficiency of free siRNA without the use of a NP is quite low, and most of the free siRNA is rapidly degraded following intravenous injection. Therefore, to overcome these limitations, we suggested the use of NP system labeled with a tumor endothelial cell-associated ligand, HA, to target the CD44 receptor. Our HA-CH-NP/siRNA delivery system can enhance selective delivery efficiency and protect siRNA from degradation during blood circulation. Additionally, we used chitosan nanoparticles (CH-NP) as a polymer matrix, which allowed strong electrostatic interactions with negatively charged siRNA. Moreover, CH has distinct characteristics such as biodegradability, biocompatibility, and low immunogenicity (Han et al., 2010). Although the NP systems have been developed, selective targeted delivery was needed for increased therapeutic benefit (Perez-Herrero & Fernandez-Medarde, 2015; Swain et al., 2016). Although a number of NP systems have been utilized for therapeutic applications, most of these were widely distributed in the body and could lead to undesirable toxicities in normal tissues (Buzea et al., 2007). In addition, wide drug distribution may require higher doses for achieving gene silencing in the target cells. Therefore, to overcome these limitations against conventional passive delivery, targeted delivery is highly desirable.

In summary, we have developed HA-labeled HA-CH-NPs/siRNA as a potential selective carrier system for PLXDC1 siRNA. PLXDC1, a cell surface transmembrane protein, is overexpressed in tumor endothelial cells, which can contribute to tumor angiogenesis, metastasis, migration, and invasion (Zhang et al., 2015). Additionally, we demonstrated a NP-based siRNA platform for tumor anti-angiogenic therapy by effective silencing of the target gene. Overall, HA-CH-NPs/PLXDC1 siRNA is a highly selective delivery system for siRNAs, which can utilize anti-angiogenic tumor therapy.

## Conclusions

Our findings may serve as a proof-of-concept for CD44 receptor-targeted delivery of HA-CH-NP/siRNA to tumor endothelial cells for anti-angiogenesis tumor therapy. Moreover, we demonstrated that PLXDC1 is an attractive therapeutic target for anti-angiogenic therapy. Herein, we suggest that the HA-CH-NP/siRNA system is an innovative and promising carrier platform that improves siRNA delivery to tumor endothelial cells. This may provide an important foundation for clinical applications of NP-based drug delivery systems. HA-CH-NP/siRNA has great potential for broad applications in anti-angiogenesis tumor therapy.

## Supplementary Material

Supplemental Material

## References

[CIT0001] Aagaard L, Rossi JJ (2007). RNAi therapeutics: principles, prospects and challenges. Adv Drug Deliv Rev 59:75–86. 17449137 10.1016/j.addr.2007.03.005 PMC1978219

[CIT0002] Bergers G, Benjamin LE (2003). Tumorigenesis and the angiogenic switch. Nat Rev Cancer 3:401–10. 12778130 10.1038/nrc1093

[CIT0003] Buzea C, Pacheco II, Robbie K (2007). Nanomaterials and nanoparticles: sources and toxicity. Biointerphases 2:MR17–71. 20419892 10.1116/1.2815690

[CIT0004] Chen C, Zhou JL, Han X, et al. (2014). A prodrug strategy based on chitosan for efficient intracellular anticancer drug delivery. Nanotechnology 25:255101. 24896540 10.1088/0957-4484/25/25/255101

[CIT0005] Chen HX, Cleck JN (2009). Adverse effects of anticancer agents that target the VEGF pathway. Nat Rev Clin Oncol 6:465–77. 19581909 10.1038/nrclinonc.2009.94

[CIT0006] Cho SH, Noh YW, Cho MY, Lim YT (2016). An electrostatically self-assembled ternary nanocomplex as a non-viral vector for the delivery of plasmid DNA into human adipose-derived stem cells. Molecules 21:572. 10.3390/molecules21050572 PMC6273813 27136523

[CIT0007] Cook KM, Figg WD (2010). Angiogenesis inhibitors: current strategies and future prospects. CA Cancer J Clin 60:222–43. 20554717 10.3322/caac.20075 PMC2919227

[CIT0008] Daka A, Peer D (2012). RNAi-based nanomedicines for targeted personalized therapy. Adv Drug Deliv Rev 64:1508–21. 22975009 10.1016/j.addr.2012.08.014

[CIT0009] Devi GR (2006). siRNA-based approaches in cancer therapy. Cancer Gene Ther 13:819–29. 16424918 10.1038/sj.cgt.7700931

[CIT0010] Fuchs B, Mahlum E, Halder C, et al. (2007). High expression of tumor endothelial marker 7 is associated with metastasis and poor survival of patients with osteogenic sarcoma. Gene 399:137–43. 17560052 10.1016/j.gene.2007.05.003 PMC2066185

[CIT0011] Griffioen AW, Coenen MJ, Damen CA, et al. (1997). CD44 is involved in tumor angiogenesis; an activation antigen on human endothelial cells. Blood 90:1150–9. 9242547

[CIT0012] Han HD, Mangala LS, Lee JW, et al. (2010). Targeted gene silencing using RGD-labeled chitosan nanoparticles. Clin Cancer Res 16:3910–22. 20538762 10.1158/1078-0432.CCR-10-0005 PMC2912984

[CIT0013] Katas H, Alpar HO (2006). Development and characterisation of chitosan nanoparticles for siRNA delivery. J Control Release 115:216–25. 16959358 10.1016/j.jconrel.2006.07.021

[CIT0014] Lallana E, Rios De La Rosa JM, Tirella A, et al. (2017). Chitosan/hyaluronic acid nanoparticles: rational design revisited for RNA delivery. Mol Pharm 14:2422–36. 28597662 10.1021/acs.molpharmaceut.7b00320

[CIT0015] Lam JK, Chow MY, Zhang Y, Leung SW (2015). siRNA versus miRNA as therapeutics for gene silencing. Mol Ther Nucleic Acids 4:e252. 26372022 10.1038/mtna.2015.23 PMC4877448

[CIT0016] Layzer JM, Mccaffrey AP, Tanner AK, et al. (2004). In vivo activity of nuclease-resistant siRNAs. RNA 10:766–71. 15100431 10.1261/rna.5239604 PMC1370566

[CIT0017] Lu C, Han HD, Mangala LS, et al. (2010). Regulation of tumor angiogenesis by EZH2. Cancer Cell 18:185–97. 20708159 10.1016/j.ccr.2010.06.016 PMC2923653

[CIT0018] Lu KV, Chang JP, Parachoniak CA, et al. (2012). VEGF inhibits tumor cell invasion and mesenchymal transition through a MET/VEGFR2 complex. Cancer Cell 22:21–35. 22789536 10.1016/j.ccr.2012.05.037 PMC4068350

[CIT0019] Ma S, Pradeep S, Hu W, et al. (2018). The role of tumor microenvironment in resistance to anti-angiogenic therapy. F1000Res 7:326. 29560266 10.12688/f1000research.11771.1 PMC5854986

[CIT0020] Mangala LS, Zuzel V, Schmandt R, et al. (2009). Therapeutic targeting of ATP7B in ovarian carcinoma. Clin Cancer Res 15:3770–80. 19470734 10.1158/1078-0432.CCR-08-2306 PMC2752981

[CIT0021] Naito M, Azuma R, Takemoto H, et al. (2017). Multilayered polyion complexes with dissolvable silica layer covered by controlling densities of cRGD-conjugated PEG chains for cancer-targeted siRNA delivery. J Biomater Sci Polym Ed 28:1109–23. 28278046 10.1080/09205063.2017.1301775

[CIT0022] Nasti A, Zaki NM, De Leonardis P, et al. (2009). Chitosan/TPP and chitosan/TPP-hyaluronic acid nanoparticles: systematic optimisation of the preparative process and preliminary biological evaluation. Pharm Res 26:1918–30. 19507009 10.1007/s11095-009-9908-0

[CIT0023] Perez-Herrero E, Fernandez-Medarde A (2015). Advanced targeted therapies in cancer: drug nanocarriers, the future of chemotherapy. Eur J Pharm Biopharm 93:52–79. 25813885 10.1016/j.ejpb.2015.03.018

[CIT0024] Ragelle H, Vandermeulen G, Preat V (2013). Chitosan-based siRNA delivery systems. J Control Release 172:207–18. 23965281 10.1016/j.jconrel.2013.08.005

[CIT0025] Ramjiawan RR, Griffioen AW, Duda DG (2017). Anti-angiogenesis for cancer revisited: is there a role for combinations with immunotherapy? Angiogenesis 20:185–204. 28361267 10.1007/s10456-017-9552-y PMC5439974

[CIT0026] Rikitake Y, Hirata K, Kawashima S, et al. (2002). Involvement of endothelial nitric oxide in sphingosine-1-phosphate-induced angiogenesis. Arterioscler Thromb Vasc Biol 22:108–14. 11788469 10.1161/hq0102.101843

[CIT0027] Sennino B, Ishiguro-Oonuma T, Wei Y, et al. (2012). Suppression of tumor invasion and metastasis by concurrent inhibition of c-Met and VEGF signaling in pancreatic neuroendocrine tumors. Cancer Discov 2:270–87. 22585997 10.1158/2159-8290.CD-11-0240 PMC3354652

[CIT0028] Seo SH, Han HD, Noh KH, et al. (2009). Chitosan hydrogel containing GMCSF and a cancer drug exerts synergistic anti-tumor effects via the induction of CD8+ T cell-mediated anti-tumor immunity. Clin Exp Metastasis 26:179–87. 19082918 10.1007/s10585-008-9228-5

[CIT0029] Swain S, Sahu PK, Beg S, Babu SM (2016). Nanoparticles for cancer targeting: current and future directions. Curr Drug Deliv 13:1290–302. 27411485 10.2174/1567201813666160713121122

[CIT0030] Urban-Klein B, Werth S, Abuharbeid S, et al. (2005). RNAi-mediated gene-targeting through systemic application of polyethylenimine (PEI)-complexed siRNA in vivo. Gene Ther 12:461–66. 15616603 10.1038/sj.gt.3302425

[CIT0031] Van Cutsem E, Tabernero J, Lakomy R, et al. (2012). Addition of aflibercept to fluorouracil, leucovorin, and irinotecan improves survival in a phase III randomized trial in patients with metastatic colorectal cancer previously treated with an oxaliplatin-based regimen. J Clin Oncol 30:3499–506. 22949147 10.1200/JCO.2012.42.8201

[CIT0032] Verheul HM, Pinedo HM (2007). Possible molecular mechanisms involved in the toxicity of angiogenesis inhibition. Nat Rev Cancer 7:475–85. 17522716 10.1038/nrc2152

[CIT0033] Weis SM, Cheresh DA (2011). Tumor angiogenesis: molecular pathways and therapeutic targets. Nat Med 17:1359–70. 22064426 10.1038/nm.2537

[CIT0034] Xu B, Lefringhouse J, Liu Z, et al. (2017). Inhibition of the integrin/FAK signaling axis and c-Myc synergistically disrupts ovarian cancer malignancy. Oncogenesis 6:e295. 28134933 10.1038/oncsis.2016.86 PMC5294249

[CIT0035] Yan H, Che X, Lv Q, et al. (2017). Grifolin induces apoptosis and promotes cell cycle arrest in the A2780 human ovarian cancer cell line via inactivation of the ERK1/2 and Akt pathways. Oncol Lett 13:4806–12. 28588729 10.3892/ol.2017.6092 PMC5452918

[CIT0036] Yang MH, Jong SB, Lu CY, et al. (2012). Assessing the responses of cellular proteins induced by hyaluronic acid-modified surfaces utilizing a mass spectrometry-based profiling system: over-expression of CD36, CD44, CDK9, and PP2A. Analyst 137:4921–33. 22910856 10.1039/c2an35368g

[CIT0037] Yang Y, Jing L, Li X, et al. (2017). Hyaluronic acid conjugated magnetic Prussian Blue@Quantum dot nanoparticles for cancer theranostics. Theranostics 7:466–81. 28255343 10.7150/thno.17411 PMC5327361

[CIT0038] Zhang ZZ, Hua R, Zhang JF, et al. (2015). TEM7 (PLXDC1), a key prognostic predictor for resectable gastric cancer, promotes cancer cell migration and invasion. Am J Cancer Res 5:772–81. 25973314 PMC4396023

