# Supplementary material for: Selective delivery of PLXDC1 small interfering RNA to endothelial cells for anti-angiogenesis tumor therapy using CD44-targeted chitosan nanoparticles for epithelial ovarian cancer
Source: Drug Deliv. 2018 Jun 11;25(1):1394–402. doi: 10.1080/10717544.2018.1480672 (PMC6096458; doi:10.1080/10717544.2018.1480672)
Supplement: Supplemental Material [file IDRD_A_1480672_SM7681.DOC]

*<Supplementary Information>*

**Selective delivery of PLXDC1 small interfering RNA to endothelial cells for anti-angiogenesis tumor therapy using CD44-targeted chitosan nanoparticles for epithelial ovarian cancer**

Ga Hee Kim ^a,k^, Ji Eun Won ^a,k^, Yeongseon Byeon ^a^, Min Gi Kim ^a^, Yun-Yong Park ^b,c^, Tae In Wi ^a^, Jae Myeong Lee ^a^, Jeong-Won Lee ^d^, Tae Heung Kang ^a^, In Duk Jung ^a^, Byung Cheol Shin ^e^, Hyung Jun Ahn ^f^, Young Joo Lee ^g^, Anil K. Sood ^h,i,j,*^, Hee Dong Han ^a^,**, and Yeong-Min Park ^a^,***

^a^Department of Immunology, School of Medicine, Konkuk University, Chungju 380-701, South Korea

^b^Asan Institute for Life Sciences, Asan Medical Center and ^c^Department of Convergence Medicine, University of Ulsan College of Medicine, Seoul, Republic of Korea

^d^Department of Obstetrics and Gynecology, Samsung Medical Center, Sunkyunkwan University School of Medicine, Seoul 06531, South Korea

^e^Bio/Drug Discovery Division, Korea Research Institute of Chemical Technology, Daejeon 305-600, South Korea

^f^Center for Theragnosis, Biomedical Research Institute, Korea Institute of Science and Technology, Seoul 136-791, South Korea

^g^Department of Bioscience and Biotechnology, Sejong University, Kwang-Jin-Gu, Seoul 143-747, South Korea

^h^Department of Gynecologic Oncology and Reproductive Medicine, ^i^Department of Cancer Biology and ^j^Center for RNA Interference and Non-coding RNA, The University of Texas M.D. Anderson Cancer Center, Texas, USA

^k^These authors contributed equally to this work

Running title: selective delivery of PLXDC1 siRNA for antiangiogenesis therapy

Keywords: chitosan nanoparticles, PLXDC1 siRNA, angiogenesis therapy, ovarian cancer

Conflict of interest: The authors declare no competing financial interests.

**Corresponding Author**

*Anil K. Sood, Department of Gynecologic Oncology and Reproductive Medicine, The University of Texas M.D. Anderson Cancer Center, Texas, USA. Tel: 713-745-5266. Fax: 713-792-3643, E-mail: [asood@mdanderson.org](mailto:asood@mdanderson.org)

**Hee Dong Han: School of Medicine, Konkuk University, 268 Chungwondae-Ro, Chungju, Chungcheong-Buk-Do 380-701, South Korea, Phone: 82-2-2049-6330, Fax: 82-2-2049-6192, E-mail: [hanhd@kku.ac.kr](mailto:hanhd@kku.ac.kr)

***Yeong-Min Park: Department of Immunology, School of Medicine, Konkuk University, 268 Chungwondae-Ro, Chungju, Chungcheong-Buk-Do 380-701, South Korea, Phone: 82-2-2049-6330, Fax: 82-2-2049-6192, E-mail: [immun3023@kku.ac.kr](mailto:immun3023@kku.ac.kr)


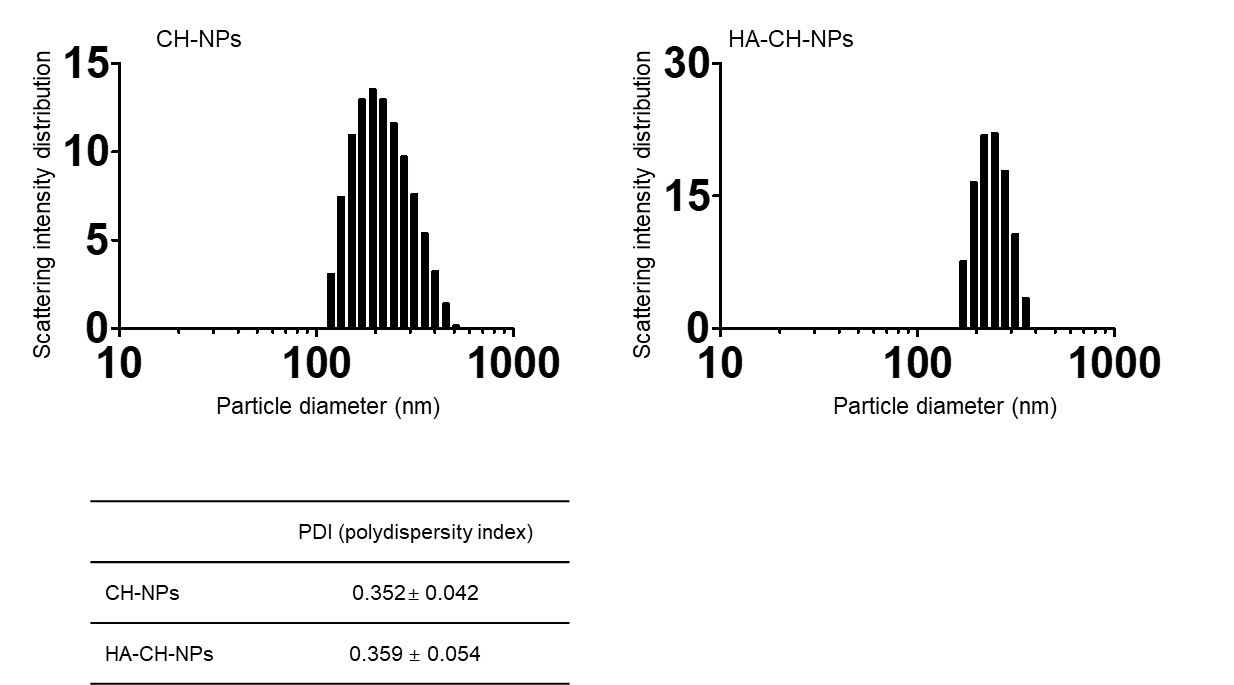


**Supplementary Figure S1.** Representative histogram of size distribution and polydispersity index for CH-NPs and HA-CH-NPs.


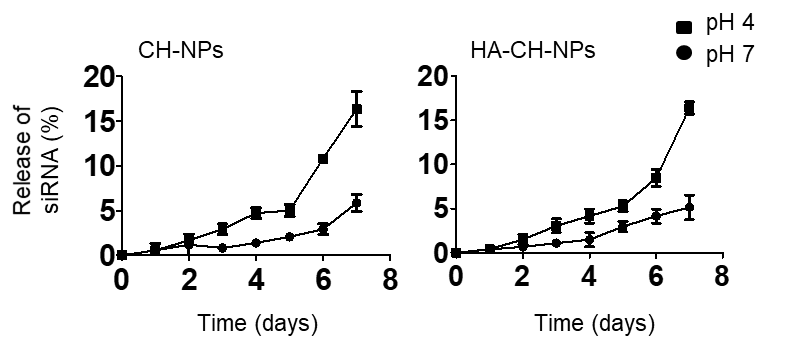


**Supplementary Figure S2.** Release of siRNA from CH-NPs or HA-CH-NPs at pH4 or pH7.


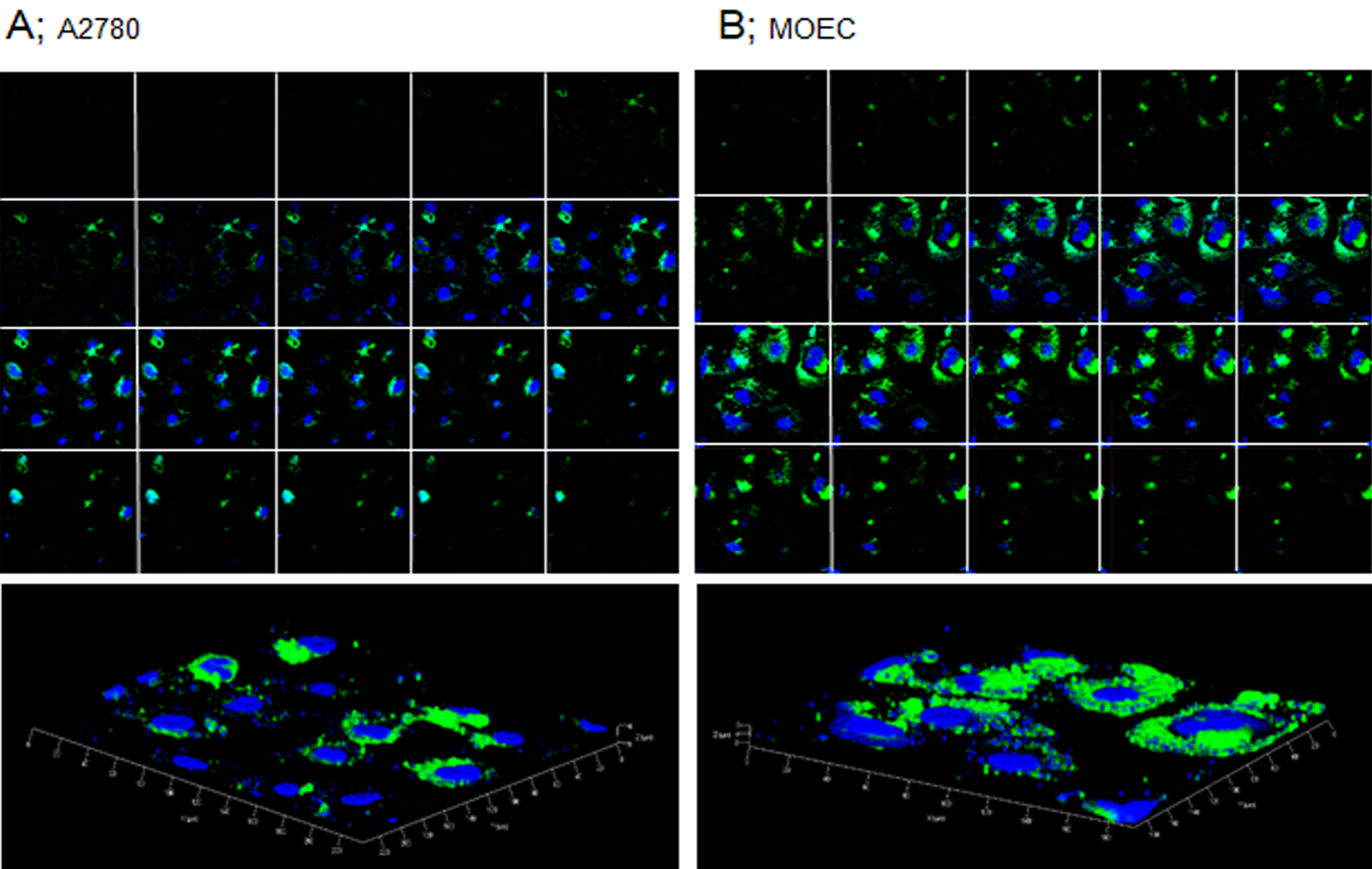


**Supplementary Figure S3.** Z-stack image of confocal microscopy for intracellular uptake of CH-NPs or HA-CH-NP (0.5 µm interval).


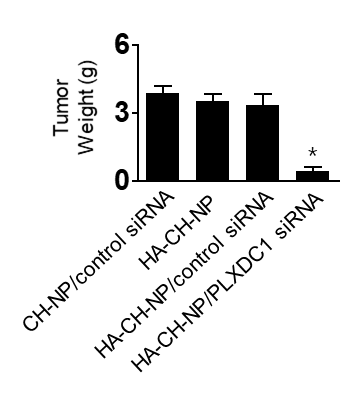


**Supplementary Figure S4.** Therapeutic efficacy of HA-CH-NP/PLXDC1 siRNA in A2780 tumor model. Treatment with HA-CH-NPs was started 1 week after the intraperitoneal (i.p.) injection of mice with A2780 tumor cells. (1) CH-NP/control siRNA as a control, (2) HA-CH-NP, (3) HA-CH-NP/control siRNA, and (4) HA-CH-NP/PLXDC1 siRNA were injected i.v. twice per week. Results represent the mean ± standard deviation (SD). Statistical tests were two-sided and p values were evaluated by analysis of variance (ANOVA). *p < 0.001


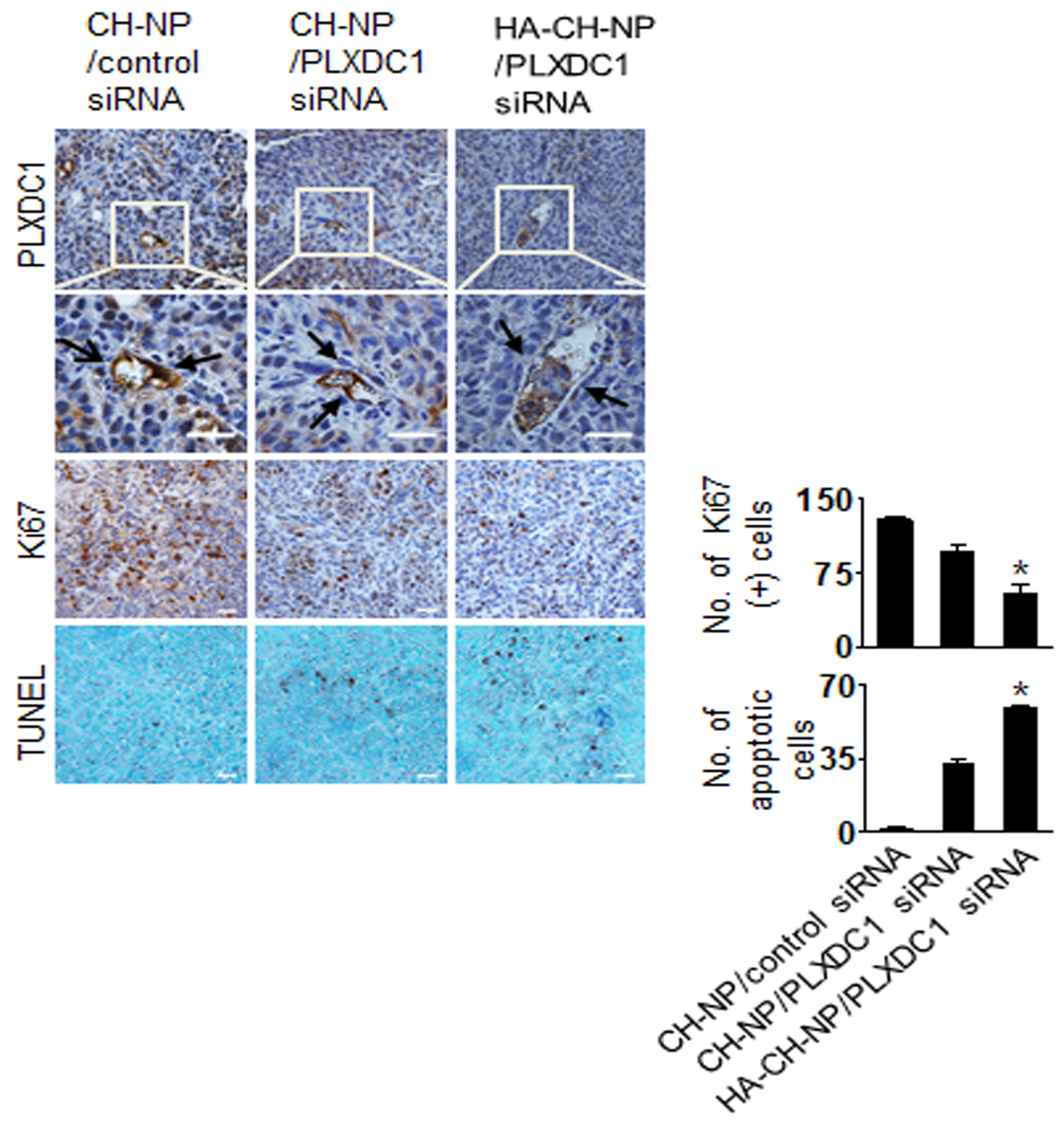


**Supplementary Figure S5.** Immunohistochemical analyses of markers of PLXDC1 expression in endothelial cells (PLXDC1 antibody), cell proliferation (Ki67), and TUNEL were performed on HeyA8 tumor tissues (scale bar: 10 µm). Error bars represent SEM. *p < 0.05.
